# Perspective: Obesity—an unexplained epidemic

**DOI:** 10.1093/ajcn/nqac075

**Published:** 2022-04-23

**Authors:** Dariush Mozaffarian

**Affiliations:** Friedman School of Nutrition Science & Policy, Tufts University, Boston, MA, USA

**Keywords:** obesity, nutrition, policy, epidemiology, prevention

## Abstract

Since 1980, obesity prevalence among US adults has soared from 14% to 42%. The commonly accepted explanation is pervasive overeating: ever-increasing energy intake as the population gains weight, year after year. However, evidence does not support this hypothesis. National data on energy intake and energy availability show increases between 1961 and 2000, during modern industrialization of food; but a plateau or declines thereafter—even as obesity continued rising—and while physical activity modestly increased. Thus, Americans appear to be eating relatively less since 2000, for ever-increasing body sizes, as time has progressed. Although both energy intake and energy availability are measured with error, such errors would have to be new since 2000 and systematically increasing over time for these 2 separate, independent measures. Given the tremendous societal consequences of obesity, and failure to date of energy balance–focused interventions to stem the tide, it is critical for the scientific community to consider and test alternative hypotheses. Growing evidence suggests complex, interrelated biological interactions between food processing (including acellular nutrients, depleted prebiotics, additives), gut microbial composition and function, host metabolic expenditure, and intergenerational transmission of risk (including epigenetics, noncoding RNAs, microbial species). In this paradigm, whereas increasing energy intake may have contributed to rising obesity in earlier years, today pervasive adiposity and its physiologic adaptations have created a biological milieu which interacts with industrialized foods to promote escalating obesity, even with stable energy intake—a self-sustaining, difficult-to-reverse cycle. These scientific hypotheses must be rigorously evaluated, because even partial confirmation would dramatically shift and expand current prevention and treatment strategies. Urgent new investment in research is required. Simultaneously, uncertain evidence on the obesity epidemic's primary drivers does not mean there is no evidence on actions that can help, and existing science must be more rapidly translated and refined into clinical, public health, and policy interventions.

## Introduction

It is time to share a striking, and not widely appreciated, secret: we do not have a clear explanation for the obesity epidemic. Since 1980, obesity prevalence among US adults has soared from 14% to 42%—a marked and unprecedented increase. Yet, the underlying reasons for this epidemic are less well understood than commonly supposed.

The widely accepted explanation—indeed, considered axiomatic—is pervasive overeating. In other words, we gain weight year after year because we eat more and more calories, year after year, as a nation. And, because each successive increment in weight leads to a new, higher steady-state of required energy, the corollary is that this overeating escalates each year: however much weight we gain, we continue to increase our energy intake even further.

This introduces a second question, of course, as to *why* our energy intake is continuously and inexorably rising. Like the notion that the obesity epidemic is caused by overeating, the explanation for this overeating is also considered axiomatic: the food environment is driving excess intake, owing to intensive marketing of hyperpalatable, widely available, inexpensive foods that activate brain reward, craving, and overeating, overpowering our biological cues for satiety in an ever-spiraling crescendo of excess.

The latter explanations as primary drivers of increasing energy intake are increasingly questioned, owing to limited empirical evidence to support their pre-eminence as well as competing evidence that overeating may at least partly be a result of obesity, rather than its cause; and that the obesity epidemic may be primarily driven by changes in diet quality (1, 2). However, the core hypothesis—that obesity is associated with increasing total energy intake each year—remains relatively unquestioned. It is important to consider the evidence supporting this hypothesis that, as nations become more obese, energy intake continues to climb, year after year.

## US Trends in Energy Intake

**FIGURE 1:**
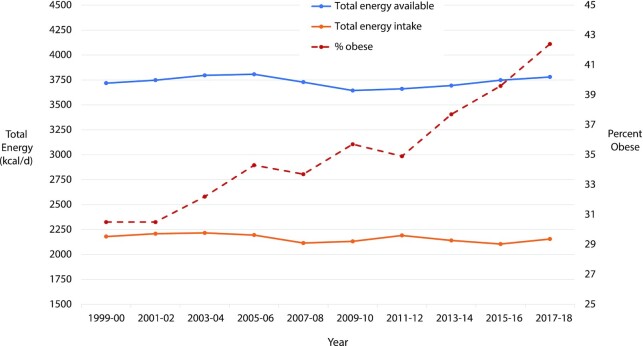
Trends in total energy consumed, total energy available, and prevalence of obesity among US adults, 1999–2018. Energy intake is based on 24-h dietary recall data from NHANES; energy availability, on national per capita FAO food balance sheets; and prevalence of obesity, on direct measurements in NHANES. FAO estimates are based on the total production, imports, and exports of foods, adjusting for foods fed to livestock, used for seed, used for manufacturing of items not for human consumption, and losses during storage and transportation. National per capita energy availability is expected to be higher than energy intake owing to usual losses from food preparation and waste. National per capita energy availability over this period has not significantly changed, whereas per capita energy calorie consumption has actually slightly decreased (*P*-trend = 0.002 based on linear regression of the mean energy consumption across each survey cycle as an ordinal variable). Because FAO reports annual values, the 2-y mean was used to correspond to NHANES data. FAO values before 2013 were adjusted for minor changes in 2014 in FAO methodology to estimate the national population, thus maintaining consistent methods in all years. Effects of this adjustment were minor; e.g., 1999 estimates were adjusted from 3673 to 3677 kcal per capita.

The United States has perhaps the best data in the world to address this question, including repeated, nationally representative surveys with standardized, interviewer-administered 24-h diet recalls in the NHANES; and accurate information on national food availability compiled and estimated by the UN FAO. Surprisingly, these data do not show any increase in energy consumption or availability over ≥20 y, a time period when obesity has steadily risen (**Figure 1**). Actually, NHANES data suggest small but statistically significant *declines* in energy intake over this period. Findings are similar when stratified by weight status (i.e., normal weight, overweight, obesity), and also similar among US children, in whom obesity rates have also steadily risen (3).

Thus, rigorously collected national data provide no support for higher energy consumption as a driver of the obesity epidemic since 2000. Strikingly, these data do not even support increasing energy consumption as a consequence of the obesity epidemic, i.e., that as people gain weight, they need to eat more. Rather, the data suggest that Americans have been eating relatively less, for their larger body sizes, over the last 2 decades.

To maintain objectivity in science, it is imperative to recognize this discordance between theory and evidence, and consider potential explanations. One explanation could be that national data on energy consumption are measured with such large errors, and that these errors are also systematically increasing over time, so that increasing trends cannot be detected. It is known that 24-h diet recalls underreport calorie intake by 8%–30%, compared to estimated energy expenditure using doubly labeled water; and that underreporting can be larger in adults with overweight or obesity (4). Thus, it is plausible that total energy intake in the NHANES is underreported, especially in people with higher weights. However, such underreporting should not suppress the detection of increasing trends over time, especially across a large national sample, because even if individuals with obesity underreport their intake, their overall mean weight has continued to increase over time. Thus, unless the magnitude of this underreporting has also systematically increased over time, their (underreported) energy intake should still have increased. In addition, a completely separate, independent measure of energy availability, from FAO food balance sheets, shows consistent findings. And, as I will discuss further, earlier trends in both energy intake and energy availability *do* show prior increases before 2000, indicating these methods can detect population trends. Together, the data do not support a hypothesis of changing energy intakes or changing reporting biases over time. Thus, although systematically increasing bias in *both* reported energy intake and national energy availability since 2000 is theoretically possible, this must be considered an explanation of exclusion. Before accepting this as truth, new empiric research is needed to test and confirm such a hypothesis; and alternative hypotheses must be considered as well.

As a second possibility, one could hypothesize that the obesity epidemic since 2000 has been driven by declining physical activity. However, like the notion of an overeating nation, the hypothesis of an increasingly sedentary nation is not supported by the evidence. US physical activity levels are far below optimal, but there is little evidence that this has meaningfully or steadily worsened since 1990. Trends in declining occupational activity, for example, have been offset by increasing leisure-time activity. National data representing diverse age, sex, race, ethnicity, income, and education subgroups demonstrate increases in self-reported physical activity over time (5–8). Separate, independent industry data are consistent with this, showing steady growth in fitness and health club membership and revenues over the last 20 y (9). As with energy intake and availability, systematically increasing bias in self-reported physical activity is theoretically possible. But, discarding all these data because they do not fit a conventional theory of obesity would be imprudent; and other hypotheses must also be considered. Thus, although national physical activity remains suboptimal, and increasing activity could help offset obesity, there is currently little evidence that widespread continually declining physical activity is a major cause of the obesity epidemic.

## The Obesity Epidemic: Changing Biology?

If the evidence does not support progressive increases in energy intake, or progressive declines in physical activity, as drivers of the obesity epidemic, it is critical for the scientific community to consider alternative explanations. Emerging evidence hints at novel, interrelated, and complementary biological pathways related to the gut microbiome, metabolic expenditure, and intergenerational transmission of risk, each influenced by a common thread of changing diet quality.

First, it is increasingly clear that the types and quality of foods consumed interact with the composition and health of our gut microbiota to influence digestive efficiency and flux (including the location, rate, and/or completeness of nutrient digestion), relative (host compared with microbiome) nutrient utilization, host metabolic expenditure, and host adipocyte function (10). The human microbiome is estimated to consume between 7% and 22% of the caloric intake of an average adult—an amount which remains poorly quantified and which also confounds measurement of human energy expenditure (whether by indirect calorimetry or doubly labeled water) (10, 11). Thus, when any food is consumed by a person, the relative use of this energy by the person's body as opposed to their microbiome may vary depending on the type of food eaten and their microbial composition and function. Over the last 50 y, changes in crop breeding, food manufacturing, and consumer choices have led to more processed starches and sugars in the diet. Such refined “acellular” carbohydrates—lacking any natural, intact plant cellular structure—are rapidly and completely digested in the stomach and small intestine, causing a double insult of excess flux of nutrients to the host and insufficient nourishment of the gut microbiome (12, 13). Fortified, acellular proteins have also increased in modern processed foods, although with less well studied health effects (14). With increased processing of foods and greater acellular nutrients, for a similar consumed energy, more energy may be absorbed by the host and less by the microbiome. Cooking of starchy plant foods also alters gut microbial composition and function, with cooked foods producing greater gains in adiposity than raw foods, even with lower total energy intakes (15). In animal models, changes to the gut microbiome can substantially alter weight gain and obesity, without changes in total energy intake or physical activity (16, 17); and changes in both absolute and relative microbiome energy use influence host weight gain (10, 11). Such complexity also extends to different dietary fats, whose effective caloric value, for the host, may vary depending on the type of food consumed and host–microbial interactions (18). In sum, a differential partitioning of consumed energy, due to population changes in foods consumed and microbial characteristics, could be a contributor to the obesity epidemic.

Second, the intrinsic metabolic expenditure of the US population may have changed over time. Dietary factors and diet–microbiome interactions appear to alter the body's metabolic expenditure, changing heat generation by influencing brown adipose tissue function as well as “browning” of beige or brown-like adipocytes within white adipose tissue (19, 20). Animal models and limited human studies further suggest that specific dietary compounds—like capsaicin and capsinoids in chili peppers, certain polyphenols, curcumin, and epigallocatechin gallate in green tea—may activate brown adipose tissue or browning of adipocytes. Randomized trials in humans also show that differing dietary compositions can alter metabolic expenditure. For instance, compared with dietary fats, higher amounts and processing of carbohydrates can reduce metabolic expenditure after weight loss by ∼200 kcal/d, with even larger effects among individuals with elevated carbohydrate-stimulated insulin secretion (21). This could partly relate to effects of rapidly digested carbohydrates on mitochondrial energetics, which appear to lower adipose tissue mitochondrial respiration and channel electrons away from antioxidants to support energy storage, especially in individuals with adiposity (22, 23). Gut microbe–generated metabolites may also influence obesity risk, even with constant food intake, owing to changes in host circadian rhythms and energy expenditure (24). In sum, these studies show that determinants of host energy expenditure are complex and potentially influenced by both nutritional composition and diet–microbiome interactions, requiring careful investigation of underlying mechanisms and implications for obesity.

Third, the obesity epidemic may be driven by intergenerational influences. Potential pathways include maternal-to-infant transmission of microbiome species (and thereby health risk); in utero epigenetic changes caused by maternal stress, obesity, and poor diet; and inter- or transgenerational transmission of sperm or oocyte noncoding RNAs (ncRNAs). As successive generations become more obese, risk may be transmitted to the next generation that increases their susceptibility independently of energy intake. The composition of the microbiome, clearly linked to risk of obesity, is transmitted from one generation to the next (25). Both dietary changes and diet-induced microbial metabolites can also induce epigenetic changes that influence risk of weight gain and obesity (26, 27); and maternal obesity and metabolism are linked to DNA methylation and childhood obesity (28, 29). ncRNAs may also play a significant role, including microRNAs (miRNAs) and fragments of transfer RNAs (tRNAs). These ncRNAs are stable over time, influence epigenetic gene expression, and are implicated in obesity and other human diseases. For example, similarly to obesity, mental health disorders in children and adolescents have increased 2- to 3-fold since the 1990s, with substantial observed heritability that may be partly explained by inter- and transgenerational transmission of ncRNAs (30). Both poor diet quality and obesity influence levels of miRNAs and tRNAs in parents (31), which can be transmitted to their offspring and thereby regulate adipocyte function, adipogenesis, inflammation, and insulin secretion and sensitivity (32). In addition, miRNAs are present in foods and appear to be absorbed, have physiologic effects, and interact with the gut microbiome (33).

In sum, complex biological interactions between the types, quality, and processing of foods and population shifts in our gut microbiome, metabolic expenditure, and intergenerational transmission of risk may be important contributors to the ongoing obesity epidemic.

## Interplay between Environment and Biology

Such biological interactions and intergenerational transmission would be most influential in populations with widespread multigenerational obesity. On the other hand, changes in energy intake from environmental changes may have contributed importantly to the onset of the obesity epidemic before adiposity and its biological adaptations were more widespread, i.e., consistent with a more simple energy balance model.

One clear antecedent to the early obesity epidemic is the industrialization of food. This included the mid-20th-century Green Revolution, a strategy to dramatically increase global agricultural production of commodity crops, and the corresponding evolution of food processing to create shelf-stable, inexpensive, starch-rich, vitamin-fortified products (34). These efforts successfully addressed the leading nutritional concerns of the time: the fear of mass starvation owing to a soaring global population, which rose from 1.6 to 6.1 billion between 1900 and 2000; the prevailing science on endemic diseases of vitamin deficiency like pellagra, scurvy, rickets, night blindness, and others; and alarm over food-borne bacterial illness. But, this industrialization also greatly reduced biodiversity, with monocropping of highly selected, mostly starch-rich crops; and greatly intensified food processing including a rise in acellular nutrients, loss of prebiotics including diverse fibers and phytonutrients, and proliferation of additives including salt, sugars, and a myriad of other preservatives, emulsifiers, stabilizers, artificial colors, and sweeteners. These changes in food production and processing in the latter half of the 20th century could have contributed the initial population “hit,” increasing energy intake, shifting the microbiome, and disrupting the prevailing, relatively stable rates of obesity.

Consistent with this, FAO food availability data show gradual increases in national per capita calorie availability from 1961 (∼2900 kcal/d) to 1980 (∼3200 kcal/d); a steeper rise from 1980 through 2000 (∼3750 kcal/d); but then no meaningful increase thereafter. Similarly, although NHANES instruments have changed from earlier years, making direct comparisons imprecise, calorie intake among adults was estimated to be ∼250 kcal/d lower in both 1971–1975 and 1976–1980 than in 1999–2000 (35). And, data from other national surveys support a gradual increase in energy intake between 1977–1978 and 1994–1996 (36). But, as obesity spread and was passed on, generation after generation, population changes in the gut microbiome, metabolic expenditure, and intergenerational transmission of risk could have become more widespread. Thus, even with a stabilizing of energy intake, obesity continued to increase. A toxic food environment begat a toxic biological environment, creating a self-sustaining, difficult-to-reverse cycle.

These possibilities require far more scientific inquiry and evidence to be established. The interplay between persistent changes in our environment and persistent changes in our biology has not been sufficiently investigated nor quantified. This lack of adequate attention and investment in understanding the root causes of the obesity epidemic—one of the most rapid and widespread alterations of human health in history—may at least partly owe to the belief that the foundational causes are already known. Ultimately, at the adipocyte cellular level, a positive energy balance must be present—but this may be influenced by biological root causes that partition energy, alter cellular function, and influence metabolic expenditure, rather than a primary imbalance of total dietary energy and total physical activity. The evidence in the United States suggests that over the last 20 y we are not eating more calories, nor exercising less, but are still becoming more obese—and that we have not elucidated, nor consequently addressed, the underlying physiology.

## Conclusions and Next Directions

The current evidence has several important implications. First, a broad acknowledgment of the paucity of evidence to support overeating as a current cause of the obesity epidemic is critical to stimulate urgent, substantial research investment to investigate other, less appreciated potential causes. Biological drivers related to the gut microbiome, metabolic expenditure, and intergenerational risk are not under direct volitional control, and will be poorly addressed by conventional educational, behavioral, product reformulation, or policy approaches focused on calories and energy balance (“eat less, move more”). If we do not understand the actual drivers, we cannot design or implement effective strategies to address them. Such work could be led, for example, by a new National Institute of Nutrition at the NIH, which could prioritize and harmonize research on this and other pressing scientific questions of our time (37).

Second, such research must consider both common and divergent causes across nations and cultures. Steady increases in energy intake could be a primary driver of obesity in populations in early stages of adiposity; whereas large reductions in physical activity, for example owing to rapid urbanization, could explain more of the obesity epidemic in certain nations, like China. On the other hand, although reliable data on calorie consumption and physical activity are far less available internationally, the axiom that global obesity is driven by urbanization is not supported by empirical evidence: direct anthropometric measurements in 112 million adults indicate that 55% of the global rise in adiposity—and >80% in some low- and middle-income regions—has been due to increased adiposity in rural areas (38).

Third, the complexity of obesity does not reduce the responsibilities of the food sector—agricultural, food manufacturer, retail, and restaurant—because industrialization of our food appears to be a core contributor to harmful biological adaptations. It is incumbent on the food sector, as well as government and foundation research funders, to deeply invest in science to more clearly understand how modern agricultural and manufacturing practices are interacting with biology to foster ever-increasing obesity. Such investment can help spur evidence-based innovation that supports both better population health and economic success of the food sector.

Finally, it is important to recognize that uncertain evidence on the primary drivers of the obesity epidemic does not mean there is no evidence on interventions that can help. Controlled trials support several strategies to help reduce weight gain and obesity, including reducing sugar-sweetened beverages and television watching; avoiding more highly processed, rapidly digestible carbohydrates; increasing minimally processed foods including those higher in unsaturated fats; implementing multicomponent nutritional and lifestyle behavioral programs in health care and worksite settings; and, in appropriate patients, utilizing pharmacologic treatment or surgery. There are also many other reasons to eat a healthy diet, such as dietary priorities for prevention of cardiovascular diseases and type 2 diabetes (39), independent of body weight. This evidence must be more rapidly translated and refined into clinical, public health, and policy approaches to help reduce obesity and other lifestyle-related conditions.

It is possible that US national data on calorie intake, and independent US national data on calorie availability, were both accurate pre-2000 but are both wrong since; that the energy balance model of obesity is correct; and that ever-increasing energy intake is the primary driver of the current obesity epidemic. But, an objective review of the evidence demands more research to support these ideas, and to consider and evaluate alternative explanations. We must consider the possibility that we are eating about the same amount, per capita, as a nation today compared with 20 y ago; and that obesity continues to increase because of complex interactions between changes in food processing including acellular nutrition and microbial shifts; diet-induced alterations in metabolic expenditure; intergenerational transmission of epigenetics, ncRNA, and microbiome composition; and other, as yet unstudied pathways. These scientific hypotheses must be rigorously evaluated, because even their partial confirmation would dramatically shift and expand our current prevention and treatment strategies.

Even amidst challenges like COVID-19, the rise in obesity over the last 30 y remains one of the most singular changes in human health in our lifetimes. Yet, we know less about the primary causes of the global obesity epidemic than we should, and the resulting societal costs greatly exceed the scientific investments to date in foundational and translational research. We do not have a clear explanation for the obesity epidemic—and it is time to acknowledge, and to correct, this regrettable truth.

## Notes

Supported by National Heart, Lung, and Blood Institute, NIH grant 2R01 HL115189 (to DM). The funder had no role in study conception or design, data analysis or interpretation, manuscript drafting or edits, or the decision to submit the manuscript for publication.

Perspective articles allow authors to take a position on a topic of current major importance or controversy in the field of nutrition. As such, these articles could include statements based on author opinions or point of view. Opinions expressed in Perspective articles are those of the author and are not attributable to the funder(s) or the sponsor(s) or the publisher, Editor, or Editorial Board of *The American Journal of Clinical Nutrition*. Individuals with different positions on the topic of a Perspective are invited to submit their comments in the form of a Perspective article or in a Letter to the Editor.

Abbreviations used: miRNA, microRNA; ncRNA, noncoding RNA; tRNA, transfer RNA.
